# Fixed-time adaptive fuzzy command filtering control for a two-joint robotic manipulator with input dead zone saturation and time-varying delay

**DOI:** 10.1371/journal.pone.0347102

**Published:** 2026-04-15

**Authors:** Xianqi Cao, Hongkui Zhang, Ping Zhang

**Affiliations:** 1 School of Applied Technology, University of Science and Technology Liaoning, Anshan, China; 2 School of Electronic and Information Engineering, University of Science and Technology Liaoning, Anshan, China; 3 School of Applied Technology and Vocational, Dalian University of Science and Technology, Dalian, China; Guangdong University of Technology, CHINA

## Abstract

This paper studies an adaptive fuzzy fixed-time tracking control problem for the two-joint robotic manipulator with input deadzone saturation and time-varying delay. By employing auxiliary signals to compensate for the effects of time-varying delay, the need for the Pade approximation method is avoided, and the requirement for the input time-varying delay to be relatively small is relaxed. Fuzzy logic systems are utilized as adaptive nonlinear approximators to identify and compensate for unknown packaged nonlinear functions within the backstepping framework. In the design of the controller, command filtering technology is introduced to address the “complexity explosion” phenomenon faced by traditional backstepping techniques. In addition, the non-smooth input saturation and dead zone nonlinearities are approximated using a non-affine smooth function, then transformed into an affine form using the mean-value theorem. The proposed method effectively combines the backstepping approach with practical fixed-time stability criteria. This guarantees the boundedness of all closed-loop signals and ensures tracking errors diminish to a small range near zero within a fixed time. More importantly, the convergence time depends solely on the design parameters. The effectiveness of the theoretical results is validated through one simulation example.

## Introduction

The robotic manipulator, which is a typical dynamic system, is widely used in industrial applications. However, challenges such as model uncertainty and unknown nonlinearities often arise, potentially affecting system performance. To address these issues, fuzzy logic systems (FLSs) are employed as nonlinear approximators, often combined with adaptive backstepping methods to manage the uncertainties inherent in these complex nonlinear systems. To date, FLSs are applied in adaptive backstepping control design, resulting in numerous significant research advancements [1–7]. In [5], an adaptive fuzzy control scheme was developed utilizing FLSs to estimate the unknown uncertainties. Furthermore, the fuzzy adaptive control framework has been extended to more complex systems, such as multi-agent systems, to solve consensus and optimal regulation problems [6,7]. The repeated differentiation of virtual control signals in standard backstepping leads to a significant issue known as “complexity explosion.” Literature [8,9] explores dynamic surface control (DSC) techniques, which address the “complexity explosion” issue by integrating backstepping control strategies with command filtering methods. Further DSC modifications presented in [10,11] address filtering imperfections by implementing compensated signal architectures. Notably, the control schemes presented in [1–11] address the adaptive infinite-time tracking control problem. However, due to the strict requirements on settling time during many real-world systems, finite-time control (FTC) has recently gained attention, and related research outcomes are gradually increasing [12–17]. For example, [12] discusses the FTC algorithm for induction motors with input saturation. Similarly, [17] proposes the path tracking control method for unmanned surface vehicles. Although the control algorithms in [12–17] can achieve control objectives within finite time, these schemes are challenging to apply when initial conditions are unavailable. In light of the drawback, Polyakov introduced fixed-time control in [18] as an effective alternative to FTC. Compared to FTC, fixed-time control guarantees that the setting time relies solely on the design parameters. Given the remarkable benefits of fixed-time control, numerous experts have applied it to the nonlinear systems [19–24]. For instance [22] proposed an improved Lyapunov-based framework that provides a tighter estimation of the settling time for nonlinear systems, addressing the conservatism in earlier results. Furthermore, it is worth noting that the tracking control problem has also been extensively studied for flexible manipulators modeled by partial differential equations (PDEs), which primarily focus on vibration suppression and boundary stabilization [25–27]. While these PDE-based studies provide a rigorous framework for distributed parameter systems, rigid multi-joint manipulators modeled by ordinary differential equations present distinct challenges. Specifically, when ODE-based systems are simultaneously plagued by complex input nonlinearities, such as dead-zone saturation and time-varying delays, achieving fixed-time convergence remains a more intricate and pressing issue. This gap in the literature further motivates the development of the control scheme proposed in this study.

Delays can severely compromise the real-time performance of control algorithms and even destabilize the system. Hence, the research on uncertain nonlinear input delay systems is particularly important. In response, several methodologies are introduced in [28–33]. In [31] the authors compensated for the input delay by applying transformation. In [33,34], A novel Lyapunov function effectively counteracts delay-induced degradation. However, The Pade approximation, a commonly used technique, requires a sufficiently small input delay, limiting its applicability to systems with delays. Besides delay, dead zone and saturation critically impair system performance, often causing substantial degradation. When the system experiences dead zone effects, the input-output relationship becomes non-linear, which can result in reduced accuracy and slower response times. Saturation, on the other hand, can limit the range of the controller of output. Therefore, it becomes crucial to consider saturation during the controller design process. The authors of [35,36] introduce the development of a novel control scheme that addresses input dead-zone and saturation. However, this work is motivated by the challenge of fixed-time tracking control for robotic manipulators subject to concurrent input delay, saturation, and dead zone.

Building upon previous research, This study aims to develop an adaptive fuzzy fixed-time control scheme for robotic manipulators, considering input dead zone, saturation, and delay. Leveraging a synthesis of fixed-time control theory and backstepping, our design incorporates command filtering and compensatory strategies. This methodology ensures system trajectories converge within a fixed time interval while maintaining all signals bounded. Key innovations of this research are listed as follows:

i) A novel adaptive fixed-time control algorithm is proposed for uncertain robotic systems with time-varying input delay, saturation, and dead zone, ensuring that the tracking error converges to a tight set near zero within a fixed time, and eliminates the impact of input delay, saturation, and dead zone nonlinearity. Notably, the convergence time is independent of initial conditions and relies only on the design parameters.
ii) Existing methods for eliminating the effects of unknown input delays mostly rely on the Pade approximation method [28–33]. However, the Pade approximation method requires that the input delay is relatively small. This restriction, does not hold in many practical systems. Therefore, this paper proposes a novel control scheme to compensate for the input delay. The proposed strategy abandons the Pade approximation method and relaxes the requirement that the time-varying input delay be relatively small.
iii) Non-smooth nonlinearities of input saturation and dead zone are addressed by a smooth non-affine approximation, which is then rendered into an affine form employing the mean-value theorem.

The following is the organization of this paper: Section 2 is preliminary results and the problem description. Section 3 details the controller design procedure using the adaptive backstepping method. Section 4 presents the stability analysis, while Sections 5 and 6 discuss the simulation results and conclusion, respectively.

## Preliminaries and problem description

The mathematical model governing the motion of a two-joint manipulator in [37] includes input dead zone saturation and input delay and is expressed as:


$$M(q)\ddot{q}+C(q,\dot{q})\dot{q}+G(q)=u(t-\tau (t))-J^{T}(q)p(t),$$ (1)

where $q=[q_{1},q_{2}{]}^{T}$, $\dot{q}$, and $\ddot{q}\in {\mathbb{R}}^{2}$ are the angular position, velocity, and acceleration vectors, respectively. The inertia matrix is $M(q)\in {\mathbb{R}}^{2\times 2}$, and the centripetal-Coriolis matrix is $C(q,\dot{q})\dot{q}\in {\mathbb{R}}^{2}$, with $G(q)\in {\mathbb{R}}^{2}$ representing the gravity vector. The matrix *J*^*T*^(*q*) is the unknown reversible Jacobian matrix, and *p*(*t*) is the restraining force. The control input is u(t−τ(t)), with $\tau (t)=[\tau_{1}(t),\tau_{2}(t){]}^{T}$ representing the input delay.

Notation 1: The matrix $2C(q,\dot{q})\dot{q}-M(q)$ is skew-symmetric when the matrix *C* has an appropriate definition. Additionally, the matrix *M*(*q*) is symmetric and positive definite.

Denote $x_{1}=[q_{1},q_{2}{]}^{T}$ and $x_{2}=[{\dot{q}}_{1},{\dot{q}}_{2}{]}^{T},$ then, one can obtain


$$\left\{{\dot{x}}_{1}=x_{2}\;{\dot{x}}_{2}=M^{-1}\left(u(t-\tau (t))-Cx_{2}-G-J^{T}(x_{1})p(t)\right)\;y=x_{1}\right)$$ (2)

To address the input delay issue, the following system will be introduced


$$\left\{{\dot{\lambda }}_{1}=\lambda_{1}-Q_{1}\lambda_{1}\;{\dot{\lambda }}_{2}=-Q_{2}\lambda_{2}+u(t-\tau (t))-u(t)\right)$$ (3)

with $\lambda_{1}$, $\lambda_{2}$ are auxiliary signals, and *Q*_1_, *Q*_2_ are positive constants.

Remark 1: The auxiliary system (3) is designed to provide active compensation for the time-varying input delay. The core mechanism is to utilize the difference between the current control input *u*(*t*) and the delayed input u(t−τ(t)) to update the auxiliary signal λ. From a mathematical perspective, when analyzing the stability of the compensated error $z_{2}=x_{2}-{\bar{\alpha }}_{1}-\lambda_{2}$, the delayed term u(t−τ(t)) in the plant dynamics is algebraically cancelled by the corresponding term. Consequently, the delay effect is neutralized, and the tracking problem is transformed into a delay-free form. This proactive approach distinguishes it from passive robust methods.

Remark 2: Compared with the traditional Pade approximation, the proposed auxiliary system (3) offers two significant advantages: i) it avoids approximation errors, allowing for a much larger range of delay τ(t) as long as it remains bounded; ii) it is naturally applicable to time-varying delays. Theoretically, the stability of the closed-loop system is guaranteed provided that the delay variation rate satisfies $\dot{\tau }(t)<1$, a condition that is satisfied in most practical robotic applications.

Additionally, $u(t)=[u_{1}(t),u_{2}(t){]}^{T}$ is the system input, and *u*_*i*_(*t*)(*i* = 1, 2) is described as follows


$$u_{i}(t)=D(v_{i})=\left\{ \begin{array}{c} I_{N},v_{i}\leq d_{s1}\\ \frac{I_{N}}{d_{s1}-d_{s2}}(v_{i}-d_{s2}),d_{s1}<v_{i}\leq d_{s2}\\ 0,d_{s2}<v_{i}\leq d_{s3}\\ \frac{I_{P}}{d_{s4}-d_{s3}}(v_{i}-b_{s3}),d_{s3}<v_{i}\leq d_{s4}\\ I_{P},v_{i}>d_{s4} \end{array}\right.$$ (4)

where *v*_*i*_ represents the input signal of the saturation and dead zone, *d*_*s*1_ < *d*_*s*2_ < 0 < *d*_*s*3_ < *d*_*s*4_, *I*_*N*_ < 0 and *I*_*P*_ > 0 are the designed saturation parameters.

Remark 3: It is important to emphasize that multiple input constraints have been investigated in various forms. For instance, the control problems for systems with input saturation and backlash nonlinearities were studied in [38] and [39]. Compared to these existing studies, the control scheme proposed in this study has the following distinct characteristics: 1) While [38] and [39] primarily focus on backlash and saturation, this work addresses a more comprehensive set of constraints, including concurrent dead-zone, saturation, and time-varying input delays. 2) Unlike the asymptotic or finite-time results in some existing literature, our approach ensures practical fixed-time stability, where the convergence time is independent of initial conditions. 3) By integrating auxiliary signals and command filtering, the proposed controller effectively compensates for the input delay without requiring the Pade approximation, which relaxes the constraints on the delay magnitude.

As shown in (4), the nonlinear characteristics present significant challenges that are difficult to address directly during the design process. Therefore,the smooth function *g*(*v*_*i*_) acts as a continuous approximation of the dead zone and saturation functions. This approach allows for more efficient handling of these nonlinearities, with *g*(*v*_*i*_) described by the following equation:


$$\begin{array}{cc} g(v_{i})= & -\frac{I_{N}}{2}\tanh k_{1}(v_{i}-\frac{d_{s1}}{k_{1}}+h_{1})+\frac{I_{P}}{2}\tanh k_{2}(v_{i}-\frac{d_{s2}}{k_{2}}+h_{2})\\ & -\frac{I_{P}}{2}\tanh k_{1}(v_{i}-\frac{d_{s3}}{k_{1}}+h_{3})+\frac{I_{P}}{2}\tanh k_{2}(v_{i}-\frac{d_{s4}}{k_{2}}+h_{4}), \end{array}$$ (5)

in which *k*_1_,$k_{2},h_{1},h_{2}$ are positive constants and adjusting these parameters can help minimize the approximation errors.

Remark 4: The selection of the smooth function *g*(*v*_*i*_) in (5) is justified by both mathematical and physical considerations. Mathematically, its *C*^∞^ continuity avoids the singularity issues inherent in piecewise linear models during backstepping differentiation and allows the application of the Mean-Value Theorem for stability analysis. Physically, it provides a more accurate representation of the “soft-switching” characteristics of actual robotic actuators, where transitions between different operating regions are typically gradual due to electromagnetic and mechanical factors.

Due to this, *u*_*i*_(*t*) can be further inferred that


$$u_{i}(t)=g(v_{i})+\Upsilon (v_{i}),$$ (6)

where the bounded approximation error $\Upsilon (v_{i})$ fulfills $|\Upsilon (v_{i})|<\gamma$, where γ corresponds to an unknown constant.

Assumption 1 [40]: The reference signal *y*_*r*_ and its time derivative ${\dot{y}}_{r}$ are bounded.

Assumption 2 [41]: There are unknown positive parameters $d_{sl},d_{su},d_{rl},d_{su},$ such that


$$0\;<\;d_{sl}<\frac{I_{N}}{d_{s1}-d_{s2}}<d_{su}<\infty \;0\;<\;d_{rl}<\frac{I_{P}}{d_{s4}-d_{s3}}<d_{ru}<\infty$$ (7)

Assumption 3 [42]: The derivative of the smooth function $g(v)=[g_{1}(v),g_{2}(v)]$ is bounded, and satisfies


$$0<m_{1}<{\dot{g}}_{j}(v)<m_{2},j=1,2$$ (8)

where *m*_1_ and *m*_2_ are positive constants

Remark 5: The reasonableness of Assumptions 1–3 is justified as follows. Assumption 1 is a standard requirement for tracking control, as any physical reference trajectory (e.g., the desired joint angle) for a robotic manipulator must stay within the robot’s finite workspace and be smooth enough for the actuators to follow. Assumption 2 is based on the physical limitations of robotic actuators. In practice, any actuator has a finite start-up torque (dead-zone) and a maximum output torque (saturation). These physical bounds, although unknown, must be finite. Assumption 3 ensures that the gain of the approximated actuator function is strictly positive and finite, which implies the controllability of the system and is a common prerequisite for applying the Mean-Value Theorem in backstepping design.

Lemma 1(Practical fixed-time stability) [43]: The nonlinear practical system $\dot{\varkappa }=f(\varkappa )$ is fixed-time stable, as long as $\beta_{1},\beta_{2},a_{1},a_{2}$ are positive constants with 0 < *a*_3_ < 1, *a*_4_ > 1 and 0<Λ<∞ such that


$$\dot{V}(\varkappa )\leq -\beta_{1}V(\varkappa {)}^{a_{3}}-\beta_{2}V(\varkappa {)}^{a_{4}}+\Lambda$$ (9)

and the settling time $T\leq \frac{1}{\beta_{2}\rho_{0}(a_{4}-1)}+\frac{1}{\beta_{1}\rho_{0}(1-a_{3})},\rho_{0}\in (0,1).$

Lemma 2 [44]: Suppose F(£) is a smooth function over the compact set Ω. For any constants δ>0, the FLSs $W^{T}\phi (\text{£})$ exist such that


$$\underset{\text{£}\in \Omega_{\text{£}}}{\sup}|F(\text{£})-W^{T}\phi (\text{£})|\leq \delta$$

in which $W=[s_{1},...,s_{q}]$ is an ideal weight vector, and the basis function vector $\phi (\text{£})=[\psi_{1},...,\psi_{q}{]}^{T}/\sum_{\sigma }^{q}\psi_{\sigma }(\text{£}),$ with $\psi_{\sigma }(\text{£})=\exp[-(\text{£}-v_{\sigma }{)}^{T}(\text{£}-v_{\sigma })/\vartheta_{\sigma }^{2}],$ $v_{\sigma }=[v_{\sigma ,1},...,v_{\sigma ,q}{]}^{T}$ being the center vector, and $\vartheta_{\sigma }$ is the width of the Gaussian function. In addition, the fuzzy approximation error δ satisfies $|\delta |\leq \delta^{*},$ in which $\delta^{*}$ is unknown constant.

Lemma 3 [45]: For ${\text{£}}_{1},$ ${\text{£}}_{2}\in \mathbb{R},$ then it follows that


$$|{\text{£}}_{1}{|}^{n_{1}}|{\text{£}}_{2}{|}^{n_{2}}\leq \frac{n_{1}n_{3}}{n_{1}+n_{2}}|{\text{£}}_{1}{|}^{n_{1}+n_{2}}+\frac{n_{2}n_{3}^{-\frac{n_{1}}{n_{2}}}}{n_{1}+n_{2}}|{\text{£}}_{2}{|}^{n_{1}+n_{2}}$$ (10)

where $n_{1}>0,n_{2}>0$ and *n*_3_ > 0 are constants.

Lemma 4 [46]: For ${ℑ}_{i}\in \mathbb{R},i=1,2,\cdots ,\rho ,$ where ℘ satisfying 0<℘<1, so that


$$(\sum_{i=1}^{\rho }|{ℑ}_{i}|{)}^{℘}\leq \sum_{i=1}^{\rho }|{ℑ}_{i}{|}^{℘}\leq \rho^{1-℘}\sum_{i=1}^{\rho }|{ℑ}_{i}{|}^{℘}$$ (11)

Lemma 5 [47]: For ${ℑ}_{j}\in \mathbb{R},k=1,2,\cdots ,\mu ,$ and ℵ satisfying ℵ>1, therefore it can be derived that


$$\sum_{k=1}^{\mu }|{ℑ}_{k}{|}^{ℵ}\leq (\sum_{k=1}^{\mu }|{ℑ}_{k}|{)}^{ℵ}\leq \mu^{ℵ-1}\sum_{k=1}^{\mu }|{ℑ}_{k}{|}^{ℵ}$$ (12)

This paper presents a fixed-time adaptive control scheme that ensures the tracking error converges to and stabilizes within a bounded vicinity of zero, with all system signals remaining uniformly bounded.

## Adaptive tracking controller design

This study develops the tracking controller for model (1) via a backstepping approach and FLSs methods, and the coordinate transformations are defined as follows:


$$\begin{array}{cc} z_{1} & =x_{1}-y_{r}-\lambda_{1}\\ z_{2} & =x_{2}-{\bar{\alpha }}_{1}-\lambda_{2} \end{array}$$ (13)

where $z_{1}=[z_{11},z_{12}{]}^{T},$ ${\bar{\alpha }}_{1}$ denotes the command filter’s output.


$$\zeta_{1}{\dot{\alpha }}_{1}+{\bar{\alpha }}_{1}=\alpha_{1},{\bar{\alpha }}_{1}(0)=\alpha_{1}(0)$$ (14)

$\alpha_{1}$ is the virtual controller with design parameter $\zeta_{1}>0$.

Remark 6: The boundedness of the command filtering error ${\bar{\alpha }}_{1}-\alpha_{1}$ is a critical prerequisite for the stability of the closed-loop system. Mathematically, this is ensured by the fact that the virtual control law $\alpha_{1}$ is a *C*^1^ function whose derivative ${\dot{\alpha }}_{1}$ remains bounded on the compact set defined by the Lyapunov stability analysis and the boundedness of reference signals in Assumption 1.

Step 1: By applying (4) and (13), ${\dot{z}}_{1}$ can be expressed as


$${\dot{z}}_{1}=x_{2}-{\dot{y}}_{r}-{\dot{\lambda }}_{1}$$ (15)

The compensating signal *q*_1_ serves to counteract filter errors


$${\dot{q}}_{1}=-k_{1}q_{1}q_{1}^{T}q_{1}+{\overset{―}{\alpha }}_{1}-\alpha_{1}+q_{2}-l_{1}th(q_{1})$$ (16)

with $th(q_{1})=[\tanh(q_{11}),\tanh(q_{12}){]}^{T},q_{1}(0)=[0,0{]}^{T},$ and *l*_1_ > 0 are design parameters.

The compensated tracking errors are denoted $s_{1}=z_{1}-q_{1},s_{2}=z_{2}-q_{2}$. One can obtain


$${\dot{s}}_{1}=s_{2}+\lambda_{2}-{\dot{y}}_{r}+\alpha_{1}+k_{1}q_{1}q_{1}^{T}q_{1}+l_{1}th(q_{1})-{\dot{\lambda }}_{1}$$ (17)

Choose the Lyapunov function as


$$V_{1}=\frac{1}{2}s_{1}^{T}s_{1}$$ (18)

From (17) and (18), the time derivative of *V*_1_ gives


$${\dot{V}}_{1}=s_{1}^{T}(s_{2}+\lambda_{2}-{\dot{y}}_{r}+\alpha_{1}+k_{1}q_{1}q_{1}^{T}q_{1}+l_{1}th(q_{1})-{\dot{\lambda }}_{1})$$ (19)

According to Lemma 4, the following inequality is easily obtained


$$s_{1}^{T}s_{2}\leq \frac{1}{2}s_{1}^{T}s_{1}+\frac{1}{2}s_{2}^{T}s_{2}$$ (20)

$$s_{1}^{T}l_{1}th(q_{1})\leq \frac{1}{2}s_{1}^{T}s_{1}+l_{1}^{2}.$$ (21)

Design the virtual controller $\alpha_{1}$


$$\alpha_{1}=-a_{1}s_{1}s_{1}^{T}s_{1}-b_{1}s_{1}^{\frac{7}{9}}+{\dot{y}}_{r}-k_{1}q_{1}q_{1}^{T}q_{1}-s_{1}+{\dot{\lambda }}_{1}-\lambda_{2}$$ (22)

in which *a*_1_, *b*_1_, and *k*_1_ are positive constants.

Substituting (20)–(22) into (19), ${\dot{V}}_{1}$ is given by


$${\dot{V}}_{1}\leq s_{1}^{T}(-a_{1}s_{1}s_{1}^{T}s_{1}-b_{1}s_{1}^{\frac{7}{9}})+\frac{1}{2}s_{2}^{T}s_{2}+l_{1}^{2}.$$ (23)

Step 2: The derivative of *z*_2_ is computed as


$${\dot{z}}_{2}={\dot{x}}_{2}-{\dot{\bar{\alpha }}}_{1}-{\dot{\lambda }}_{2}.$$ (24)

The compensated errors are defined as $s_{2}=z_{2}-q_{2}$, with *q*_2_ given by


$${\dot{q}}_{2}=-k_{2}q_{2}q_{2}^{T}q_{2}-q_{1}-l_{2}th(q_{2})$$ (25)

with $th(q_{2})=[\tanh(q_{21}),\tanh(q_{22}){]}^{T},q_{2}(0)=[0,0{]}^{T},$ and *l*_2_ > 0 are design parameters.

By combining equations (3), (24) and (25), one can obtain


$${\dot{s}}_{2}=M^{-1}(Q_{2}\lambda_{2}+u(t)-Cx_{2}-G-J^{T}(x_{1})P(t))-{\dot{\bar{\alpha }}}_{1}-{\dot{q}}_{2}$$ (26)

Select the Lyapunov function candidate as:


$$V_{2}=V_{1}+\frac{1}{2}s_{2}^{T}Ms_{2}+\sum_{j=1}^{2}\frac{m_{1}}{2\eta_{2j}}{\tilde{\theta }}_{2j}^{T}{\tilde{\theta }}_{2j}$$ (27)

where $\eta_{2j}>0$ are design parameters, and ${\tilde{\theta }}_{2j}=\theta_{2j}-{\hat{\theta }}_{2j}$, with the definition of $\theta_{2j}$ provided later.

According to (26) and (27), ${\dot{V}}_{2}$ is described by


$$\begin{array}{cc} {\dot{V}}_{2}=\; & {\dot{V}}_{1}+s_{2}^{T}M(M^{-1}(u-G-J^{T}(x_{1})P(t)-{\dot{\bar{\alpha }}}_{1}+k_{2}q_{2}q_{2}^{T}q_{2}+l_{2}th(q_{2})+q_{1}))\\ & +\frac{1}{2}s_{2}^{T}\dot{M}s_{2}-s_{2}^{T}Cs_{2}-\sum_{j=1}^{2}\frac{m_{1}}{2\eta_{2j}}{\tilde{\theta }}_{2j}^{T}{\dot{\hat{\theta }}}_{2j} \end{array}$$ (28)

Based on Notation 1 and equation (6), it can be concluded that ${\dot{V}}_{2}$


$${\dot{V}}_{2}={\dot{V}}_{1}+s_{2}^{T}(g(v)+\Upsilon (v)+f_{2}(X)+k_{2}q_{2}q_{2}^{T}q_{2}+l_{2}th(q_{2}))-\sum_{j=1}^{2}\frac{m_{1}}{2\eta_{2j}}{\tilde{\theta }}_{2j}^{T}{\dot{\hat{\theta }}}_{2j}$$ (29)

in which $f_{2}(X)=-G-J^{T}(x_{1})P(t)+Q_{2}\lambda_{2}+M(k_{2}q_{2}q_{2}^{T}q_{2}+l_{2}th(q_{2})+q_{1})-k_{2}q_{2}q_{2}^{T}q_{2}+l_{2}th(q_{2})+2s_{2},$ where $X_{i}=[x_{1},x_{2},\lambda_{1},\lambda_{2},y_{r},{\dot{y}}_{r}{]}^{T}$.

Using Lemma 2, FLSs are utilized to approximate $f_{2}(X)=[f_{21},f_{22}{]}^{T},$ as follows:


$$f_{2j}=W_{2j}^{T}\phi_{2j}(X)+\varepsilon_{2j},j=1,2,$$ (30)

where $\varepsilon_{2j}$ satisfies $\varepsilon_{2j}\leq \varepsilon_{2j}^{*},$ with $\varepsilon_{2j}^{*}$ being a constant.

Based on Lemma 4, one obtains the following inequality


$$s_{2}^{T}l_{2}th(q_{2})\leq \frac{1}{2}s_{2}^{T}s_{2}+l_{2}^{2}$$ (31)

$$s_{2}^{T}\Upsilon (v)\leq \frac{1}{2}s_{2}^{T}s_{2}+\gamma^{2}$$ (32)

$$s_{2}^{T}f_{2}(X)\leq \sum_{j=1}^{2}(\frac{m_{1}s_{2j}^{2}\theta_{2j}}{2h_{2j}}\phi_{2j}^{T}\phi_{2j}+\frac{h_{2j}}{2})+\frac{s_{2}^{T}s_{2}}{2}+\sum_{j=1}^{2}\frac{(\varepsilon_{2j}^{*}{)}^{2}}{2}$$ (33)

in which $\theta_{2j}=\frac{||W_{2j}|{|}^{2}}{m_{1}},$ and *h*_2*j*_>0 are the design constants.

Putting (31)–(33) to (29), ${\dot{V}}_{2}$ is derived as


$${\dot{V}}_{2}\leq -a_{1}s_{1}^{T}s_{1}s_{1}^{T}s_{1}-b_{1}s_{1}^{T}s_{1}^{\frac{7}{9}}+s_{2}^{T}g(v)+\sum_{j=1}^{2}\frac{m_{1}s_{2j}^{2}\theta_{2j}}{2h_{2j}}\phi_{2j}^{T}\phi_{2j}-\sum_{j=1}^{2}\frac{m_{1}}{2\eta_{2j}}{\tilde{\theta }}_{2j}^{T}{\dot{\hat{\theta }}}_{2j}+\Delta_{1}$$ (34)

where $\Delta_{1}=l_{1}^{2}+l_{2}^{2}+\gamma^{2}+\sum_{j=1}^{2}(\frac{(\varepsilon_{2j}^{*}{)}^{2}}{2}+\frac{h_{2j}}{2})$

Design the virtual controller *v*


$$v=-a_{2}s_{2}s_{2}^{T}s_{2}-b_{2}s_{2}^{\frac{7}{9}}-[\frac{s_{21}^{2}{\hat{\theta }}_{21}\phi_{21}^{T}\phi_{21}}{2h_{21}},\frac{s_{22}^{2}{\hat{\theta }}_{22}\phi_{22}^{T}\phi_{22}}{2h_{22}}{]}^{T}$$ (35)

and the adaptive laws


$${\dot{\hat{\theta }}}_{2j}=\frac{\eta_{2j}}{2h_{2j}}s_{2j}^{2}\phi_{2j}^{T}\phi_{2j}-F_{2j}{\hat{\theta }}_{2j}^{3}-G_{2j}{\hat{\theta }}_{2j}$$ (36)

under the condition that $a_{2},b_{2},\eta_{2j},h_{2j},F_{2j}$ and *G*_2*j*_ are the positive parameter.s.

On account of the meanvalue theorem and Assumption 3, the following inequality holds


$$s_{2}^{T}g(v)\leq -a_{2}m_{1}s_{2}^{T}s_{2}s_{2}^{T}s_{2}-b_{2}m_{1}s_{2}^{T}s_{2}^{\frac{7}{9}}-\sum_{j=1}^{2}\frac{m_{1}s_{2j}^{2}{\hat{\theta }}_{2j}\phi_{2j}^{T}\phi_{2j}}{2h_{2j}}$$ (37)

By substituting (35)–(37) into (34), ${\dot{V}}_{2}$ can be estimated by


$$\begin{array}{cc} {\dot{V}}_{2}\leq & -a_{1}s_{1}^{T}s_{1}s_{1}^{T}s_{1}-b_{1}s_{1}^{T}s_{1}^{\frac{7}{9}}-a_{2}m_{1}s_{2}^{T}s_{2}s_{2}^{T}s_{2}-b_{2}m_{1}s_{2}^{T}s_{2}^{\frac{7}{9}}\\ & +\sum_{j=1}^{2}\frac{\rho_{1}}{\eta_{2j}}{\tilde{\theta }}_{2j}(F_{2j}{\hat{\theta }}_{2j}^{3}+G_{2j}{\hat{\theta }}_{2j})+\Delta_{1} \end{array}$$ (38)

### Stability analysis

Theorem 1: For the model (1) under Assumptions 1–3, by constructing the virtual controllers (22), the actual controllers (35), adaptive laws (36), the proposed control method guarantees that all signals remain bounded and the tracking error *z*_1_ converges to a small region near the zero within fixed-time.

Proof: Choose the whole Lyapunov function


$$V=\frac{1}{2}s_{1}^{T}s_{1}+\frac{1}{2}s_{2}^{T}Ms_{2}+\sum_{j=1}^{2}\frac{m_{1}}{2\eta_{2j}}{\tilde{\theta }}_{2j}^{T}{\tilde{\theta }}_{2j}$$ (39)

From (38) and (39), $\dot{V}$ can be represented by


$$\begin{array}{cc} \dot{V}\leq & -a_{1}s_{1}^{T}s_{1}s_{1}^{T}s_{1}-b_{1}s_{1}^{T}s_{1}^{\frac{7}{9}}-a_{2}m_{1}s_{2}^{T}s_{2}s_{2}^{T}s_{2}-b_{2}m_{1}s_{2}^{T}s_{2}^{\frac{7}{9}}\\ & +\sum_{j=1}^{2}\frac{\rho_{1}}{\eta_{2j}}{\tilde{\theta }}_{2j}(F_{2j}{\hat{\theta }}_{2j}^{3}+G_{2j}{\hat{\theta }}_{2j})+\Delta_{1} \end{array}$$ (40)

On account of the fact that


$$\sum_{j=1}^{2}\frac{m_{1}F_{2j}}{\eta_{2j}}{\tilde{\theta }}_{2j}{\hat{\theta }}_{2j}^{3}=\sum_{j=1}^{2}\frac{m_{1}F_{2j}}{\eta_{2j}}({\tilde{\theta }}_{2j}\theta_{2j}^{3}-3\theta_{2j}^{2}{\tilde{\theta }}_{2j}^{2}+3{\tilde{\theta }}_{2j}^{3}\theta_{2j}-{\tilde{\theta }}_{2j}^{4})$$ (41)

$$\sum_{j=1}^{2}\frac{m_{1}F_{2j}}{\eta_{2j}}3{\tilde{\theta }}_{2j}\theta_{2j}^{3}\leq \sum_{j=1}^{2}\frac{m_{1}F_{2j}}{\eta_{2j}}(3{\tilde{\theta }}_{2j}^{2}\theta_{2j}^{2}+\frac{\theta_{2j}^{4}}{12})$$ (42)

$$\sum_{j=1}^{2}\frac{m_{1}F_{2j}}{\eta_{2j}}3{\tilde{\theta }}_{2j}^{3}\theta_{2j}\leq \sum_{j=1}^{2}\frac{m_{1}F_{2j}}{\eta_{2j}}(\frac{9{\overset{―}{\lambda }}^{3}{\tilde{\theta }}_{2j}^{4}}{4}+\frac{\theta_{2j}^{4}}{4\overset{―}{\lambda }})$$ (43)

where $\overset{―}{\lambda }>0$ is the design constant. Substituting (42) and (43) to (41), the following inequality holds that


$$\sum_{j=1}^{2}\frac{m_{1}F_{2j}}{\eta_{2j}}{\tilde{\theta }}_{j}{\hat{\theta }}_{j}^{3}\leq \sum_{j=1}^{2}\frac{m_{1}F_{2j}}{\eta_{2j}}(\frac{(9{\overset{―}{\lambda }}_{j}^{\frac{4}{3}}-4){\tilde{\theta }}_{j}^{4}}{4}+\frac{({\overset{―}{\lambda }}_{j}^{4}+9)\theta_{j}^{4}}{12{\overset{―}{\lambda }}_{j}^{4}})$$ (44)

Combining (40) and (44), one has


$$\begin{array}{cc} \dot{V}\leq & -a_{1}(s_{1}^{T}s_{1}{)}^{2}-b_{1}\sum_{j=1}^{2}s_{1j}^{\frac{16}{9}}-a_{2}m_{1}(s_{2}^{T}s_{2}{)}^{2}-b_{2}m_{1}\sum_{j=1}^{2}s_{2j}^{\frac{16}{9}}-\sum_{j=1}^{2}\frac{m_{1}F_{2j}}{\eta_{2j}}(\frac{(4-9{\overset{―}{\lambda }}^{\frac{4}{3}}){\tilde{\theta }}_{2j}^{4}}{4})\\ & +\sum_{j=1}^{2}\frac{m_{1}G_{2j}}{\eta_{2j}}{\tilde{\theta }}_{2j}{\hat{\theta }}_{2j}-\sum_{j=1}^{2}m_{1}(\frac{G_{2j}{\tilde{\theta }}_{2j}^{2}}{2\eta_{2j}}{)}^{\frac{8}{9}}+\sum_{j=1}^{2}m_{1}(\frac{G_{2j}{\tilde{\theta }}_{2j}^{2}}{2\eta_{2j}}{)}^{\frac{8}{9}}+\Delta_{2} \end{array}$$ (45)

where $\Delta_{2}=\Delta_{1}+\sum_{j=1}^{2}(\frac{m_{1}F_{2j}}{\eta_{2j}}\frac{({\overset{―}{\lambda }}^{4}+9)\theta_{2j}^{4}}{12{\overset{―}{\lambda }}^{4}}).$

Concurrently, based on Lemma 4 and let ${\text{£}}_{1}=m_{1}(\frac{G_{2j}{\tilde{\theta }}_{2j}^{2}}{2\eta_{2j}}{)}^{\frac{8}{9}},{\text{£}}_{2}=1,n_{1}=\frac{8}{9},n_{2}=\frac{1}{9}$ and *n*_3_ = 8, which yields


$$\sum_{j=1}^{2}m_{1}(\frac{G_{2j}{\tilde{\theta }}_{2j}^{2}}{2\eta_{2j}}{)}^{\frac{8}{9}}\leq \sum_{j=1}^{2}m_{1}(\frac{G_{2j}{\tilde{\theta }}_{2j}^{2}}{2\eta_{2j}}{)}^{\frac{8}{9}}+\sum_{j=1}^{2}\frac{m_{1}G_{2j}8^{8}}{9^{9}}$$ (46)

One can obtain that


$$\sum_{j=1}^{2}\frac{m_{1}G_{2j}}{\eta_{2j}}{\tilde{\theta }}_{2j}{\hat{\theta }}_{2j}\leq -\sum_{j=1}^{2}\frac{m_{1}G_{2j}}{\eta_{2j}}{\tilde{\theta }}_{j}^{2}+\sum_{j=1}^{2}\frac{m_{1}G_{2j}}{\eta_{2j}}\theta_{2j}^{2}$$ (47)

By using Lemma 5, Lemma 6 and substituting (46)–(47) into (45), $\dot{V}$ can be further represented by


$$\begin{array}{cc} \dot{V}\leq & -4a_{1}(\frac{1}{2}s_{1}^{T}s_{1})-2^{\frac{8}{9}}b_{1}(\frac{s_{1}^{T}s_{1}}{2}{)}^{\frac{8}{9}}-4a_{2}m_{1}(\frac{1}{2}s_{2}^{T}s_{2}{)}^{2}-2^{\frac{8}{9}}b_{2}m_{1}(\frac{s_{2}^{T}s_{2}}{2}{)}^{\frac{8}{9}}\\ & -\frac{\overset{―}{F}\overset{―}{\eta }(4-9{\overset{―}{\lambda }}^{\frac{4}{3}})}{2m_{1}}(\sum_{j=1}^{2}\frac{m_{1}}{2\eta_{2j}}{\tilde{\theta }}_{2j}^{2}{)}^{2}-\overset{―}{G}(\sum_{j=1}^{2}\frac{m_{1}}{2\eta_{2j}}{\tilde{\theta }}_{2j}^{2}{)}^{\frac{8}{9}}+\Delta_{3}\\ & \leq -\beta_{1}V^{\frac{8}{9}}-\beta_{2}V^{2}+\Delta_{3} \end{array}$$ (48)

with $\frac{\overset{―}{F}\overset{―}{\eta }(4-9{\overset{―}{\lambda }}^{\frac{4}{3}})}{2m_{1}}=\min\{\frac{F_{21}\eta_{21}(4-9{\overset{―}{\lambda }}^{\frac{4}{3}})}{2m_{1}},\frac{F_{22}\eta_{22}(4-9{\overset{―}{\lambda }}^{\frac{4}{3}})}{2m_{1}}\}$, $\bar{G}=\min\{G_{21},G_{22}\},\beta_{2}=\min\{4a_{1},4a_{2}m_{1},\frac{\overset{―}{F}\overset{―}{\eta }(4-9{\overset{―}{\lambda }}^{\frac{4}{3}})}{2m_{1}}\},$

$\beta_{1}=\min\{2^{\frac{8}{9}}b_{1},2^{\frac{8}{9}}b_{2}m_{1},\bar{G}\}$, and $\Delta_{3}=\Delta_{2}+\sum_{j=1}^{2}\frac{m_{1}G_{2j}}{\eta_{2j}}\theta_{2j}^{2}+\sum_{j=1}^{2}\frac{m_{1}G_{2j}8^{8}}{9^{9}}.$

Remark 7: It should be noted that the use of inequality relaxations, such as Young’s inequality and Lemma 3, introduces a certain degree of conservatism in the theoretical residual set $\Delta_{3}$. This conservatism is a necessary trade-off to ensure the robustness of the two-joint robotic manipulator against lumped uncertainties and input delays. In practical implementation, the actual tracking performance is often superior to the theoretical bound because the fuzzy logic systems provide high-precision approximation, and the convergence region can be further reduced by appropriately tuning the control gains *a*_*i*_ and *b*_*i*_.

By Lemma 1, the settling time can obtain


$$T_{1}\leq \frac{1}{\beta_{1}\rho_{0}}+\frac{9}{\beta_{2}\rho_{0}},0<\rho_{0}<1.$$ (49)

It can be inferred from $s_{j}=z_{j}-q_{j}($for *j* = 1,2). Since *q*_*j*_ is bounded in fixed-time, *z*_*j*_ is consequently bounded.

Define the Lyapunov function candidate as


$$V_{q}=\frac{1}{2}q_{1}^{T}q_{1}+\frac{1}{2}q_{2}^{T}q_{2}$$ (50)

We have


$${\dot{V}}_{q}=q_{1}^{T}(-k_{1}q_{1}q_{1}^{T}q_{1}+{\overset{―}{\alpha }}_{1}-\alpha_{1}+q_{2}-l_{1}th(q_{1}))+q_{2}^{T}(-k_{2}q_{2}q_{2}^{T}q_{2}-q_{1}-l_{2}th(q_{2}))$$ (51)

Based on [48], it can be concluded that ${\overset{―}{\alpha }}_{1j}-\alpha_{1j}<c_{1j}$ and ${\overset{―}{\alpha }}_{2j}-\alpha_{2j}<c_{2j}$ in *T*_2_, in which *c*_1*j*_ and *c*_2*j*_ are known constants. Hence if *l*_1_ > *c*_1*j*_, *l*_2_ > *c*_2*j*_ then, there holds


$$\begin{array}{cc} {\dot{V}}_{q}\leq & -k_{1}q_{1}^{T}q_{1}q_{1}^{T}q_{1}-k_{2}q_{2}^{T}q_{2}q_{2}^{T}q_{2}-\sum_{i=1}^{2}\sum_{j=1}^{2}(l_{i}-c_{ij})|q_{ij}|\\ \leq & -k_{3}V_{1}^{2}-\bar{w}V_{1}^{\frac{1}{2}} \end{array}$$ (52)

where $k_{3}=4\min\{k_{1},k_{2}\},\bar{w}=\sqrt{2}\min\{l_{i}-c_{ij}\}.$ By Lemma 1, *q*_*j*_ converges to a zero vicinity within fixed-time *T*_3_. Consequently, since *z*_*j*_ = *s*_*j*_ + *q*_*j*_, the error *z*_*j*_ is also bounded within a fixed time $T\leq T_{1}+T_{2}+T_{3}$.

Remark 8: To provide a clear guideline for practical implementation and ensure system stability, the influence of design parameters and the systematic tuning procedure are summarized as follows: 1) Parameter Influence: The fixed-time gains *a*_*i*_, *b*_*i*_ primarily govern the convergence speed; larger values reduce the settling time but increase the risk of actuator saturation. The adaptive gains $\eta_{2j}$ determine the learning rate of the fuzzy logic systems, where excessive values may lead to chattering in the control input. The auxiliary gains *Q*_*i*_ control the delay compensation speed, and the filter constant $\zeta_{1}$ represents the trade-off between command-following accuracy and signal smoothness. 2) Tuning Procedure: A step-by-step tuning roadmap is recommended to avoid excessive control effort: i) Initialize *a*_*i*_, *b*_*i*_ with moderate positive values to ensure baseline stability while keeping *u*(*t*) within physical limits; ii) Adjust the filter constant $\zeta_{1}$ to eliminate potential high-frequency oscillations; iii) Gradually increase *Q*_*i*_ to neutralize the *t*racking lag caused by input delay; iv) Finally, tune the adaptive gains $\eta_{2j}$ to refine the steady-state accuracy, ensuring the leakage terms *F*_2*j*_, *G*_2*j*_ are active to maintain parameter boundedness.

## Simulation studies

In this part, a simulation of a two-DOF robotic manipulator is conducted to validate the efficacy of the proposed algorithm:


$$\left\{{\dot{x}}_{1}=x_{2}\;{\dot{x}}_{2}=M^{-1}(u(t-\tau (t)-Cx_{2}-G-J^{T}(x_{1})p(t)))\;y=x_{1}\right)$$

Select the gravitational force


$$G(q)=\left[\begin{array}{c} \left(A_{1}L_{4}+A_{2}L_{1})g\cos(q_{1})+A_{2}L_{4}g\cos(q_{1}+q_{2}\right)\\ A_{2}L_{4}g\cos(q_{1}+q_{2}) \end{array}\right]$$

Choose the inertia matrix, the reversible Jacobian matrix, and the centripetal and Coriolis torques as


$$\begin{array}{c} M(q)=\left[\begin{array}{cc} A_{2}(L_{1}^{2}+L_{4}^{2}+2L_{1}L_{4}\cos(q_{2})+A_{1}L_{3}^{2}+K_{1}+K_{2} & A_{2}(L_{4}^{2}+L_{1}L_{4}\cos(q_{2})+K_{2}\\ A_{2}(L_{4}^{2}+L_{1}L_{4}\cos(q_{2})+K_{2} & A_{2}L_{4}^{2}+K_{2} \end{array}\right] \end{array}$$

$$\begin{array}{c} J(q)=\left[\begin{array}{cc} -L_{1}\sin(q_{1})+L_{2}\sin(q_{1}+q_{2}) & L\cos(q_{1})+L_{2}\cos(q_{1}+q_{2})\\ -L_{2}\sin(q_{1}+q_{2}) & L_{2}\cos(q_{1}+q_{2}) \end{array}\right] \end{array}$$

$$\begin{array}{c} C(q,\dot{q})=\left[\begin{array}{cc} -A_{2}L_{1}L_{4}{\dot{q}}_{2}\sin({\dot{q}}_{2}) & A_{2}L_{1}L_{4}{\dot{q}}_{2}\sin({\dot{q}}_{2})\\ -A_{2}L_{1}L_{4}({\dot{q}}_{1}+{\dot{q}}_{2})\sin({\dot{q}}_{2}) & 0 \end{array}\right] \end{array}$$

The system involves two links with mass *A*_1_ = *A*_2_ = 0.7 kg, and lengths *L*_1_ = 0.36 m and *L*_2_ = 0.32 m, with *L*_3_ and *L*_4_ representing the midpoints of the respective links. The inertia of the links are $K_{1}=56.12\times 10^{-3}\;\text{kg}\cdot {\text{m}}^{2}$ and $K_{2}=19.78\times 10^{-3}\;\text{kg}\cdot {\text{m}}^{2}$, and the gravitational acceleration is *g* = 9.81 m/s^2^. Additionally, the external disturbance is defined as $p={\left[0.5\sin(t)+1.5,1.5\cos(t)+0.5\right]}^{T}$. The delays $\tau_{i}(t)$ are


$$\begin{array}{cc} \text{case 1}: & \tau_{i}(t)=0.0025+0.001\sin(t),i=1,2\\ \text{case 2}: & \tau_{i}(t)=0.07+0.004\cos(t),i=1,2 \end{array}$$

The designed parameters $d_{s1}=-25,d_{s2}=-0.5,d_{s3}=0.1,d_{s4}=28,I_{N}-25,I_{P}=30.$ Hence, the input *u* is chosen as


$$u=\left\{ \begin{array}{c} I_{N},v\leq d_{s1}\\ \frac{I_{N}}{d_{s1}-d_{s2}}(v-b_{s0}),d_{s1}<v\leq d_{s2}\\ 0,d_{s2}<v\leq d_{s3}\\ \frac{I_{N}}{d_{s4}-d_{s3}}(v-b_{s3}),d_{s3}<v\leq d_{s4}\\ I_{P},v>d_{s4} \end{array}\right.$$

Select the initial conditions to be $x_{1}(0)=[0.5,0.5{]}^{T},x_{2}(0)=[0.5,0.3{]}^{T},\theta_{2}(0)=[0.5,0.3{]}^{T},$ and the desired signal is $y_{r}={\left[\sin(2t),\cos(2t)\right]}^{T}.$ The controller parameters are selected as $a_{1}=[0.3,0.3{]}^{T},b_{1}=[15,10{]}^{T},k_{1}=[10,10{]}^{T},l_{1}=0.2;a_{2}=[0.3,0.3{]}^{T},b_{2}=[15,10{]}^{T},k_{2}=[10,10{]}^{T},l_{2}=0.2.\eta_{21}=\eta_{22}=5.2,$ $h_{21}=h_{22}=5,F_{21}=F_{22}=0.52,G_{21}=G_{22}=0.832,\zeta =[0.001,0.001{]}^{T},Q_{1}=Q_{2}=1,$ and the fuzzy membership functions are


$$\mu_{{\bar{f}}_{2}}(X_{2})=exp\left[-\frac{(X_{2}-3+L{)}^{2}}{4}\right],L=1,2,3,4,5$$

**Fig 1:**
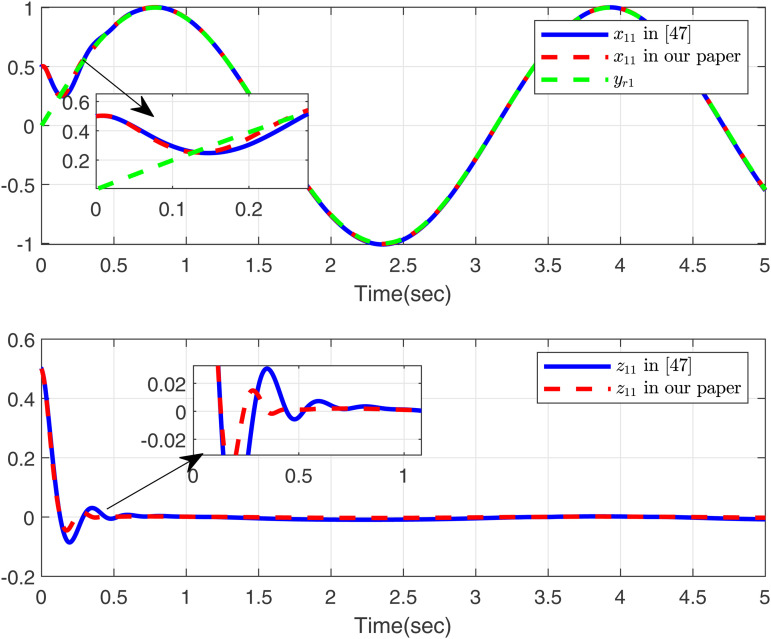
Curves of *x*_11_ and *z*_11_ in this paper and in [49], desired signal *y*_*r*1_ under case 1.

**Fig 2:**
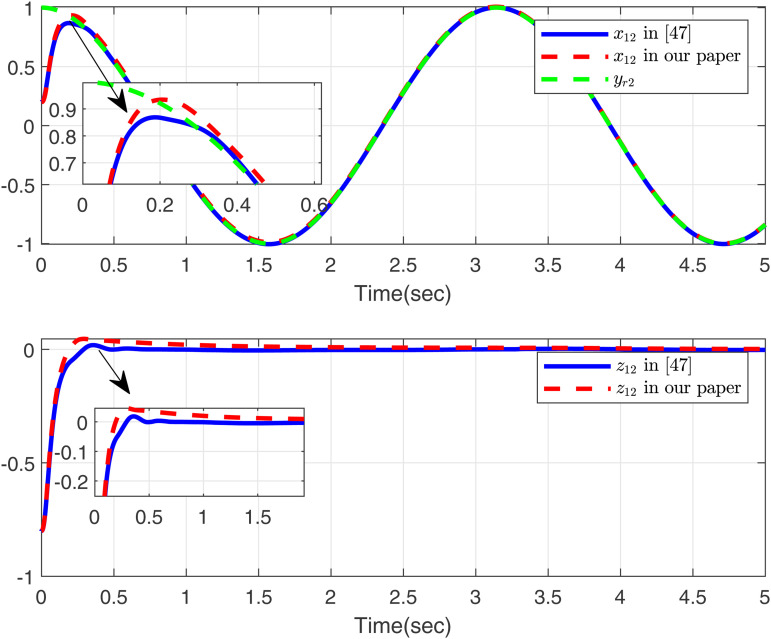
Curves of *x*_12_ and *z*_12_ in this paper and in [49], desired signal *y*_*r*2_ under case 1.

Firstly, we consider systems without input saturation and dead zone to compare with the simulation results in [49], aiming to further demonstrate the effectiveness of our method in handling time-varying delays. Then, Figs 1-2 present a comparative analysis between the proposed method and [49], with case 1 considering the time delay. As shown in Fig 1, the proposed method tracks *x*_11_, *y*_*r*1_, and *z*_11_ excellently, outperforming the approach in [49]. Fig 2 displays the trajectories of *x*_12_, *y*_*r*2_, and *z*_12_ for the proposed method and [49]. Both controllers perform effectively in the presence of time delay as modeled in case 1.

Based on the parameters chosen for the simulation, the theoretical settling time is calculated as *T*_max_ = 1.15 s. From the simulation results in Fig 1 and Fig 2, the actual settling time is approximately 0.42 s and 0.48 s, respectively, which are well within the predicted theoretical bound. This demonstrates the effectiveness of the proposed fixed-time control scheme.

**Fig 3:**
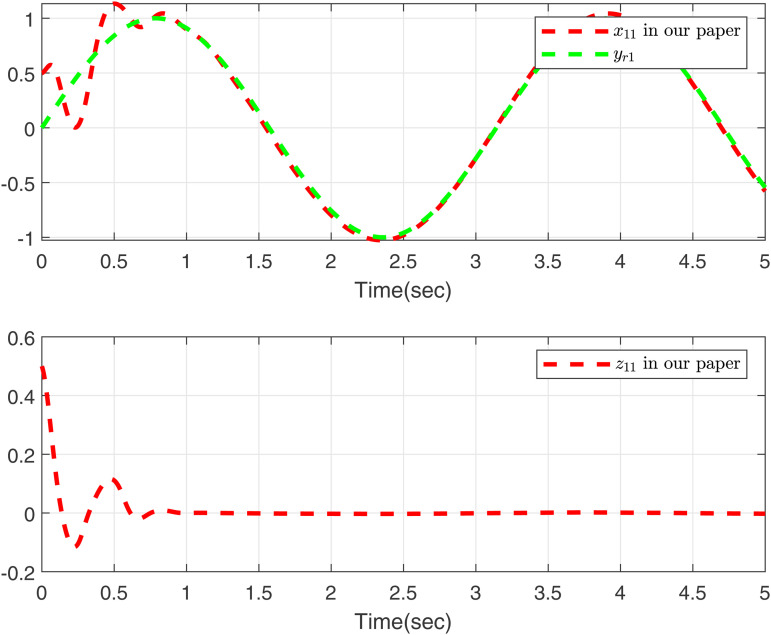
Curves of *x*_11_ and *z*_11_ in this paper, desired signal *y*_*r*1_ under case 2.

**Fig 4:**
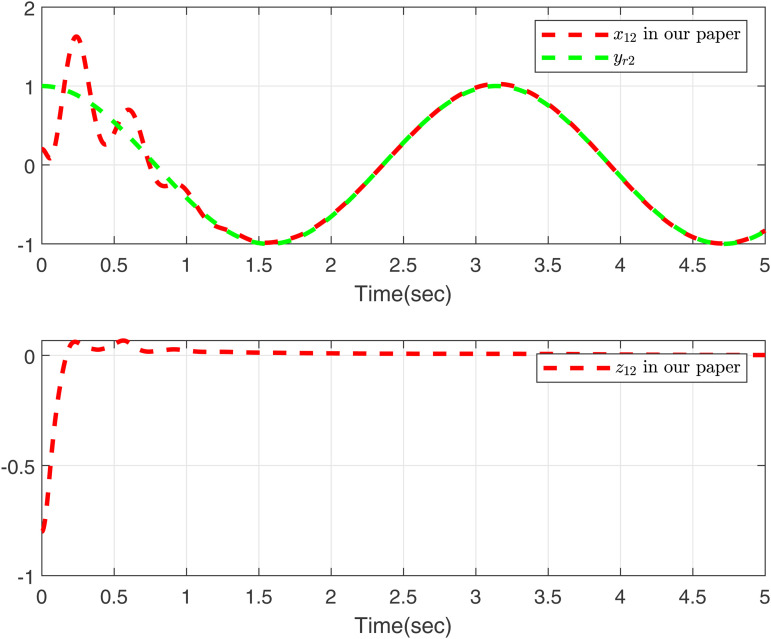
Curves of *x*_12_ and *z*_12_ in this paper, desired signal *y*_*r*2_ under case 2.

**Fig 5:**
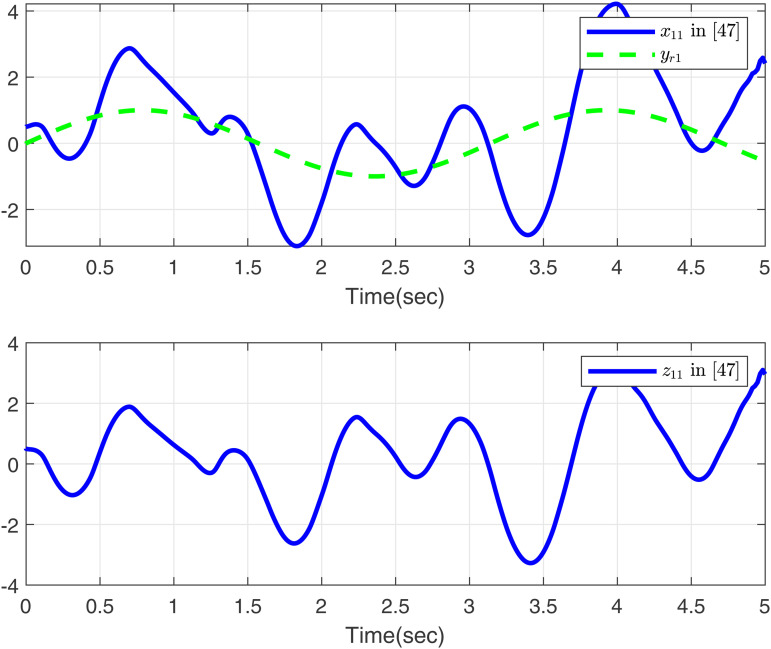
Curves of *x*_11_ and *z*_11_ in [49], desired signal *y*_*r*1_ under case 2.

**Fig 6:**
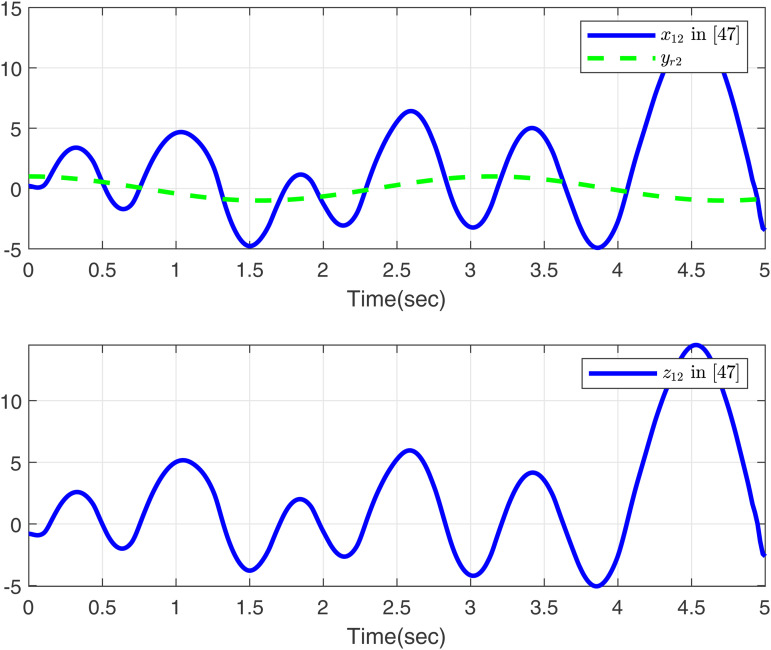
Curves of *x*_12_ and *z*_12_ in [49], desired signal *y*_*r*2_ under case 2.

Performance of the control strategies in case 2, considering delays, is evaluated in Figs 3–6. Specifically, Figs 3 and 5 compare the curves of $x_{11}$, *y*_*r*1_, and *z*_11_ for both methods, and Figs 4 and 6 compare those of *x*_12_, *y*_*r*2_, and *z*_12_. The results lead to a clear conclusion: the proposed method effectively handles the time-varying delay system in case 2, while the approach in [49] does not yield feasible control.

As shown in Figs 1–6 the method in [49] is only feasible when the delay derivative is relatively small input delay. Despite relaxing the common constraint on the magnitude of time-varying input delays, the proposed method maintains excellent tracking performance. This capability significantly enhances both the theoretical value and practical relevance of the approach.

Remark 9: The failure of the method in [49] under Case 2 is primarily due to its reliance on the Pade approximation, which is only valid for relatively small delays. In contrast, the proposed auxiliary signal compensation avoids local approximations, thus tolerating a much wider range of delay magnitudes. Moreover, even if the delay information contains small estimation errors, the adaptive FLSs can effectively compensate for the resulting residual uncertainties, ensuring the robustness of the tracking performance.

**Fig 7:**
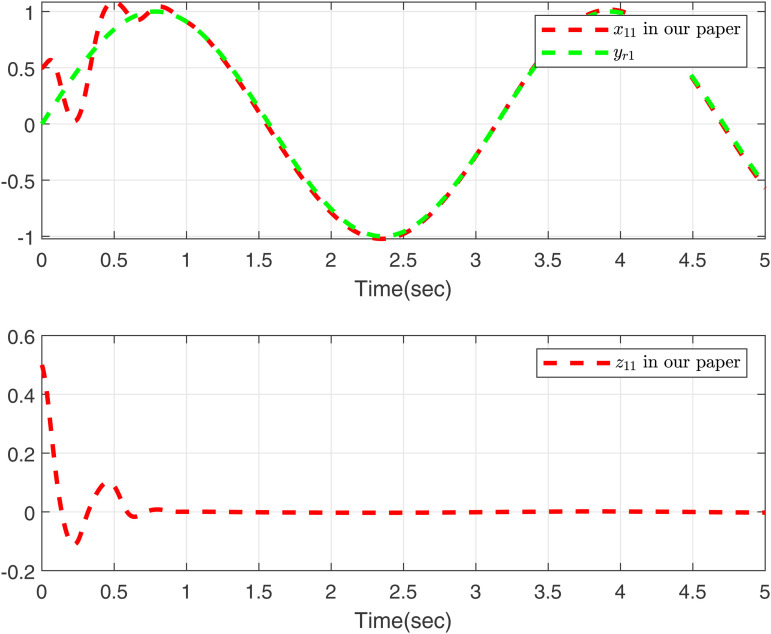
Curves of *x*_11_ and *z*_11_ in this paper, desired signal *y*_*r*1_.

**Fig 8:**
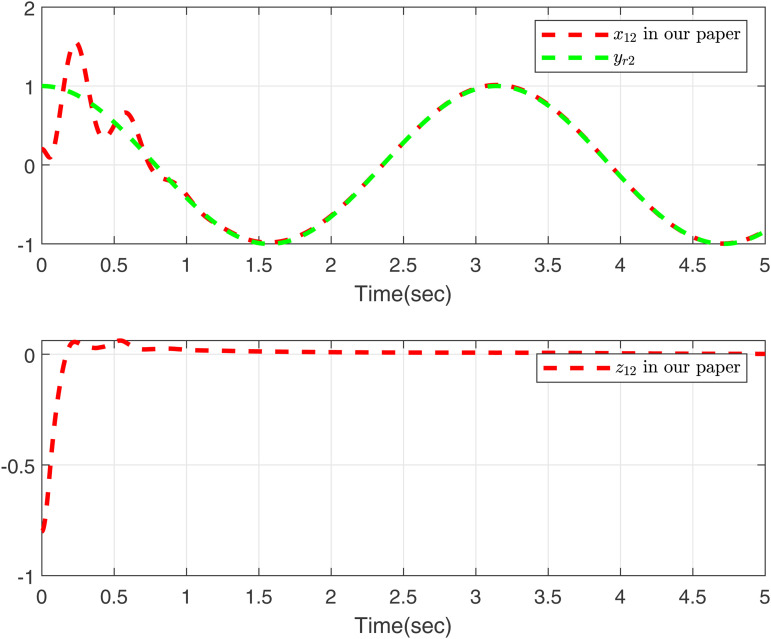
Curves of *x*_12_ and *z*_12_ in this paper, desired signal *y*_*r*2_.

**Fig 9:**
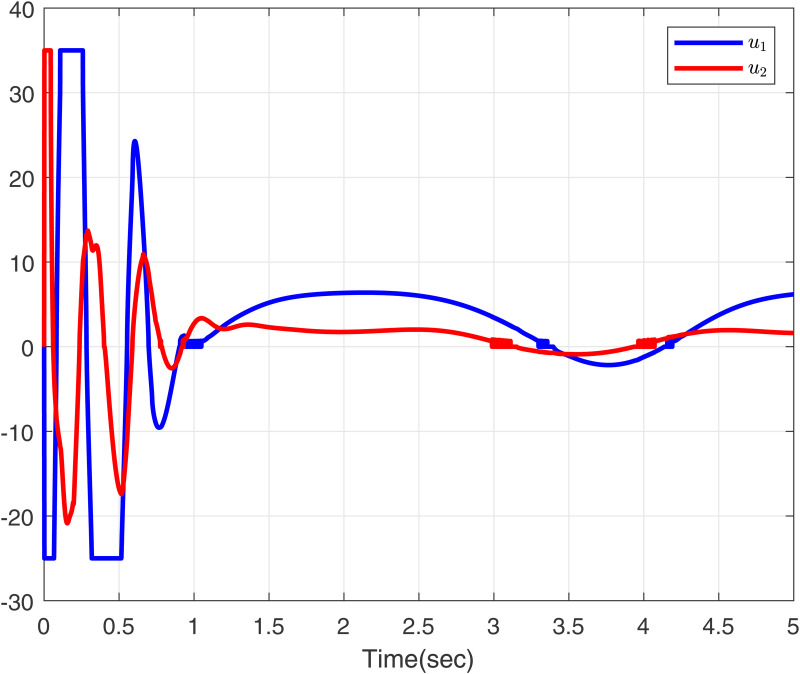
Curves of control inputs *u*_1_ and *u*_2_ in this paper.

**Fig 10:**
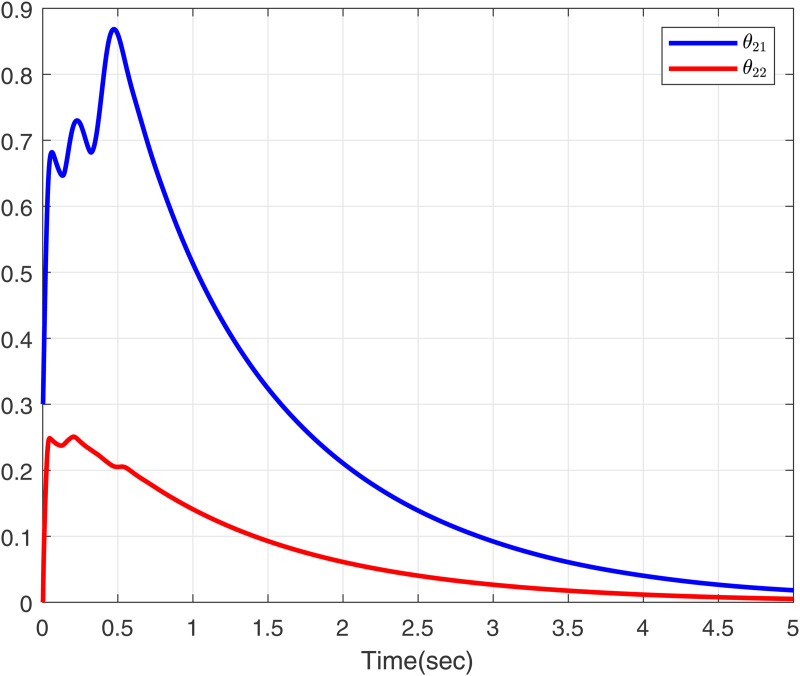
The curves of adaptive laws$\theta_{1}$ and $\theta_{2}$ in this paper.

To further validate the proposed method, we make input delay saturation and dead zone into consideration and the time-varying delays are chosen as τ(t)=0.05+0.02cos(t). Figs 7 and 8 present the curves of *x*_11_, *y*_*r*1_, and *z*_11_, and *x*_12_, *y*_*r*2_, and *z*_12_, all of which exhibit excellent tracking performance. Fig 9 shows the response trajectories of control input *u*_1_, *u*_2_ for the proposed method. Fig 10 shows the curves of adaptive laws $\theta_{1},\theta_{2}$ in this paper. The boundedness of all closed-loop signals is demonstrated in Figs 7–10.

## Conclusion

This paper addresses the problem of controlling a two-joint robotic manipulator with input dead zone, saturation, and time-varying delays by developing an adaptive fixed-time fuzzy control scheme. The method eliminates the impact on unknown time-varying input delays using auxiliary signals to compensate for time-varying input delay, thereby refraining from using the Pade approximation method and relaxes the requirement that the time-varying input delay be relatively small. By utilizing command filtering technology, the method effectively addresses the address the “complexity explosion” phenomenon. Furthermore, a non-affine smooth function approximates the input saturation and dead-zone nonlinearities. By synthesizing backstepping and fixed-time stability criteria, the proposed scheme guarantees boundedness of all closed-loop signals and drives the tracking error to a small region near zero within a fixed time, thereby making the convergence time purely parameter-dependent. In future research, we aim to extend the proposed fixed-time control framework to multi-agent robotic systems and investigate the impact of event-triggered communication to reduce the computational burden on the actuators. Furthermore, the application of this method to real hardware experiments will be conducted to further validate its practical performance.

## Supporting information

S1 File MATLAB simulation source code. This ZIP file contains the MATLAB scripts and controller models used to generate the simulation results and figures presented in this study.
(ZIP)
